# Positive Self-Perception of Aging and Quality of Life in People Living with HIV: The Role of Cultural Stereotype Internalization

**DOI:** 10.3390/healthcare14132011

**Published:** 2026-07-06

**Authors:** Ana Laguía, Antonio Bustillos, Cristina Moreno, Inmaculada Jarrín, María José Fuster-RuizdeApodaca

**Affiliations:** 1 Facultad de Psicología, Universidad Nacional de Educación a Distancia (UNED), C/Juan del Rosal, 10, 28040 Madrid, Spain; aglaguia@psi.uned.es (A.L.);; 2 Instituto Mixto de Investigación-Escuela Nacional de Sanidad (IMIENS), 28029 Madrid, Spain; 3 Centro Nacional de Epidemiología, Instituto de Salud Carlos III, 08036 Barcelona, Spain; 4 Sociedad Española Interdisciplinaria del Sida, Gta. de Quevedo, 9, 5°, Chamberí, 28036 Madrid, Spain

**Keywords:** HIV, health-related quality of life, self-perception of aging, age stereotypes

## Abstract

**Highlights:**

**What are the main findings?**

- The internalization of cultural stereotypes about older adults’ competence is shown to have a significant effect in ameliorating the negative effect of comorbidities.
- Chronological age of the sample was not related to any of the stereotypical measures, self-perception of aging, or the health events assessed retrospectively over the previous two years.

**What are the implications of the main findings?**

- These findings suggest important new avenues for interventions aimed at improving the quality of life of people living with HIV, in addition to treating comorbidities, by addressing a positive internalization of cultural stereotypes.
- A better knowledge of stereotype internalization could help design interventions aimed at both patients and their caregivers that improve the perception of aging and reduce the harmful effects of stereotypes associated with their condition as older PLHIV.

**Abstract:**

**Introduction**: Individuals aging with HIV face significant challenges that can negatively impact their long-term health, including biomedical complications, premature aging, and the influential role of aging-related stereotypes. Our study aimed to investigate whether a more positive self-perception of aging is associated with better health-related quality of life and less adverse impacts of premature aging on people living with HIV. **Methods**: We conducted a multicenter study combining a cross-sectional survey with a 2-year retrospective extraction of clinical data from medical records, involving 285 participants aged ≥ 50 years (77.9% male) living with HIV in Spain. Participants reported their health-related quality of life, self-perception of aging, and cultural age-related stereotypes. Additionally, two years of retrospective clinical health data were collected for eight areas: Metabolic, Neurological, Neoplastic, Bone, Hepatic, Renal, Cardiovascular, and Others. **Results**: As expected, worse physical health over the past two years had a negative impact on the self-perception of aging. Cultural age-related stereotypes of warmth and competence were found to predict their internalization into the self-concept. However, only self-perceived competence mediated the influence of the cultural age-related stereotype of the competence dimension on a more positive self-perception of aging. Furthermore, a positive self-perception of aging positively predicts all dimensions of health-related quality of life. **Conclusions**: Our data suggest two interrelated pathways that may influence the aging process: a clinical pathway, characterized by the negative impact of pre-existing comorbidities, and a psychosocial pathway, related to the internalization of cultural stereotypes. This study highlights the impact of cultural stereotypes on the self-perception of aging through their internalization in a sample of individuals experiencing premature aging.

## 1. Introduction

Over 40 million people are living with HIV worldwide [1]. The number of people living with HIV (PLHIV) in Spain is currently estimated to be between 140,000 and 160,000 [2]. The rate of new HIV diagnoses in Spain has been declining progressively and slowly from 8.58 per 100,000 inhabitants in 2010 to 7.44 in 2024 [2,3]. Improvements in antiretroviral therapy (ART) have been able to change AIDS from an acute infection with higher rates of mortality to a chronic condition [4]. This shift has led to increased life expectancy, and consequently, a gradual increase in the number of people aging with HIV. Findings from a large dataset of PLHIV in the Netherlands estimated that the proportion of PLHIV aged 50 years or older—an age often used as a cut-off point to define older adults in PLHIV [5]—would increase from 28% in 2010 to 73% in 2030 [6]. Moreover, these authors predicted that 84% of PLHIV would have at least one age-related non-communicable disease. Other research has suggested that this overall pattern is likely to be repeated elsewhere in Europe and North America [7]. In Spain, in 2016, 46.3% of PLHIV were 50 years or older, and the percentage of PLHIV ≥ 50 in hospital care increased from 12% in 2004 to 55.4% in 2018 [5]. In other words, the increasing life expectancy of PLHIV poses several challenges, including premature aging.

Furthermore, there is a growing consensus on the central role of improving health-related quality of life (HRQoL) in achieving long-term health outcomes for PLHIV and enhancing healthcare efficiency [8,9]. Improvements in HRQoL may also lead to improvements in other aspects, such as medication adherence, retention in care, and clinical outcomes, including better mental health, reduced morbidity, fewer hospitalizations, and lower mortality [10,11,12].

Particularly, people aging with HIV face essential challenges that can undermine their long-term health, healthy aging, and HRQoL. These challenges include clinical, treatment, and social aspects, among others. Age appears to be associated with complex changes in the immune system that may increase susceptibility to certain diseases [13]. PLHIV may be frail and could present reduced functional reserve due to an impaired and senescent immune system, several comorbid conditions, and many years of ART [4,13,14,15,16]. In addition, older people with HIV often experience psychosocial stressors, including multiple manifestations of HIV-related stigma and intersectional stigmas related to age, sexual orientation, gender, or substance use, which are considered major determinants of their HRQoL and mental health [17]. Prior research on older people with HIV has documented high levels of stigma and ageism, as well as their association with depression, social isolation, and poorer wellbeing. Experiences of stigma and discrimination can also affect self-management, social participation, and access to support, thereby intersecting with clinical factors in shaping quality of life in later life [18,19,20].

One important layered stressor that people experience as they age is age-related stereotyping [21] and the “paternalistic prejudice” stemming from feelings of compassion elicited by stereotypes about older adults. According to the Stereotype Content Model [22], older adults are culturally perceived as low in competence and high in warmth. They are often pitied rather than respected and viewed as having low status, which elicits perceptions of low competence and passivity, while reinforcing perceptions of warmth [23]. One important consequence of age stereotyping is stereotype internalization, or assimilation, as individuals grow older [24]. Research has shown that more positive self-perceptions of aging are associated with greater longevity (up to 7.5 additional years of life expectancy) and better health outcomes. Levy’s Stereotype Embodiment Theory [25] proposes that older adults tend to internalize societal stereotypes about aging, and that these self-perceptions, beliefs, and expectations about their aging process can significantly affect their health. Accordingly, negative age stereotypes are associated with poorer health outcomes, while positive age stereotypes are linked to better health among older adults.

Consistent with Stereotype Embodiment Theory, recent work with older people with HIV has shown that internalized ageist and HIV-related stereotypes are associated with poorer mental health and lower wellbeing, suggesting that self-perceptions of aging and stigma may be important pathways linking social attitudes to health outcomes [18,19]. Older people with HIV are therefore exposed not only to ageist stereotypes that portray older adults as frail, dependent and less competent, but also to HIV-related stereotypes that depict them as irresponsible, threatening, or socially marginalized. The convergence of these stigmas can reinforce perceptions of vulnerability and dependence and may make older PLHIV particularly sensitive to cultural stereotypes of aging and to their internalization into self-views.

Together, the clinical and psychosocial factors create a demanding context in which older PLHIV’s thoughts and feelings about their own aging may become particularly relevant to their HRQoL. Self-perception of aging may therefore play a relevant role, as previous research has shown that the inflammatory process mediates the association between positive self-perceptions of aging and longevity, and that these inflammatory pathways can be related to psychosocial stressors [21]. In this context, past experiences with clinical comorbidities may also shape psychological self-perceptions of aging. The accumulation of non-AIDS clinical events (NAEs) over time can make experiences of physical decline, functional limitation, and medical vulnerability more salient, potentially reinforcing negative expectations about aging and strengthening the internalization of age-related stereotypes into the self-concept. From the perspective of the Stereotype Embodiment Theory, repeated encounters with illness and disability may act as concrete episodes through which cultural beliefs about frailty and decline are confirmed or challenged, thereby influencing how older PLHIV evaluate their own aging process [25,26].

Overall, previous research has shown that HRQoL in PLHIV is influenced by a combination of clinical factors (such as comorbidities, treatment burden, and immune aging) and psychosocial stressors (including stigma, ageism, and social disadvantages) [13,14,15,16]. HRQoL in PLHIV has therefore been widely studied, and existing work has documented the impact of comorbidities, treatment burden, and stigma on patient-reported outcomes [17,18,19,20]. However, the role of aging-related self-perceptions and culturally shared age stereotypes remains underexplored, particularly in the Spanish context, where research on age stereotypes and HIV has been scarce. Spain offers a particularly relevant context for studying these issues because of its rapid demographic ageing of the HIV population, its high levels of ART uptake and viral suppression, and the increasing emphasis on integrating HRQoL into national HIV plans [2,3]. Understanding how aging-related beliefs and self-views relate to HRQoL in this setting may therefore provide insights that are informative for other high-income health systems facing similar epidemiological and policy challenges.

In line with Wurm and colleagues [26], we understand cultural age stereotypes (or meta-stereotypes) as the shared beliefs in a society about how older adults are typically perceived [22,27]. When older adults apply these cultural beliefs to themselves, they engage in self-stereotyping [28]. Self-perception of aging reflects individuals’ subjective evaluations and expectations about their own aging, shaped by their behavioral experiences, personality, and life history [24,25]. Although these constructs are related, in this study cultural stereotypes refer to socially shared views about older adults, internalized stereotypes refer to the degree to which these views are incorporated into the self-concept, and self-perception of aging refers to broader personal evaluations of one’s own aging process. Building on the Stereotype Content Model [22], we focus specifically on the competence and warmth dimensions of cultural age-related stereotypes and on their internalization into self-stereotypes among older adults living with HIV. In line with the Stereotype Embodiment Theory [25], we conceptualize self-perception of aging as a key psychological mechanism by which internalized age-related beliefs may influence health and HRQoL. Accordingly, our conceptual model links cultural age stereotypes, self-stereotypes of competence and warmth, self-perception of aging, non-AIDS comorbidities, and different domains of HRQoL as interrelated components of the aging process in PLHIV.

### The Present Research

The main objective of the present study was to examine the role of self-perception of aging in the HRQoL of adults aged 50 years and older living with HIV in Spain. Specifically, we aimed to: (1) analyze whether NAEs are associated with a less positive self-perception of aging; (2) investigate how cultural age-related stereotypes and their internalization (self-stereotypes of competence and warmth) relate to self-perception of aging; and (3) test whether self-perception of aging is associated with different domains of HRQoL. Based on the Stereotype Embodiment Theory, we hypothesized that a more positive self-perception of aging would be associated with better HRQoL and would partly account for the link between NAEs and HRQoL. By jointly modelling clinical comorbidities, cultural age stereotypes, internalized stereotypes, and self-perception of aging, this study seeks to address the limited evidence on how aging-related beliefs and self-views contribute to HRQoL among older PLHIV, particularly in the Spanish context, and to provide insights that may inform more person-centered and age-inclusive models of HIV care.

## 2. Materials and Methods

### 2.1. Study Design and Participants

We conducted a multicentre observational study combining a cross-sectional survey with a 2-year retrospective review of clinical data in the CoRIS cohort. This cohort is an open, prospective, multicentre cohort of adults with HIV in Spain that has enrolled ART-naive participants since 2004.

According to predefined inclusion criteria, CoRIS coordinators at each participating hospital generated a list of potentially eligible patients aged 50 years or older who were already receiving routine HIV care in Galicia, Madrid, Catalonia, Aragon, Andalusia and Valencia. Eligibility criteria included having an HIV-positive diagnosis, being aged ≥ 50 years, receiving ART, having no severe psychiatric or cognitive disorder according to clinical records, and providing written informed consent.

During routine medical consultations or when attending other hospital services, healthcare professionals explained the study’s goals to these eligible patients and invited them to participate. Those who agreed completed the paper-based survey containing the self-reported measures on their own in the waiting rooms or other designated spaces within the hospital and returned the questionnaire to the healthcare professionals. Healthcare providers were available to clarify doubts but did not remain with participants while they answered, and no identifying information was included in the survey to minimize potential social desirability pressures, even though participation was offered by the treating clinicians.

A total of 394 people living with HIV were recruited. An a priori target sample size of approximately 400 participants was established based on national estimates of the prevalence of people living with HIV aged 50 years or older and the proportion of new HIV diagnoses in this age group at the time of the study. This sample size was considered adequate for the planned PLS-SEM analyses, given the complexity of the model and recommended minimum ratios of cases to parameters. For the present analyses, we included only those participants with at least two years of retrospective clinical data in the CoRIS database, resulting in a final sample of 285 individuals. The overall response rate was around 95%, with centre-specific rates ranging from approximately 88% to 100%. The most commonly reported reasons for declining participation were lack of time to complete the questionnaire during the visit or a preference not to take part in the study at that moment.

For each participant, clinical data were extracted retrospectively from the CoRIS database for the 24 months preceding the questionnaire completion date, which constituted the cross-sectional assessment point of the study. We also evaluated the feasibility of using a 3-year retrospective window. However, this option substantially reduced the sample size because some participants had entered the cohort more recently and some centres had incomplete reporting for earlier years. Therefore, the 2-year window was selected as a clinically meaningful and methodologically feasible period for capturing recent clinical markers and non-AIDS events (NAEs).

### 2.2. Measures

#### 2.2.1. Validated Self-Reported Measures

The following validated self-reported measures were included in the cross-sectional survey:

Health-related Quality of Life (HRQoL) was assessed using the Spanish version of the WHOQOL-HIV-BREF [29]. This scale comprises 31 items, two about general health and 29 covering six domains: physical health; psychological health; level of independence; social relations; environmental health; and spirituality, religion, and personal beliefs (SRPB) [30]. Responses to all items are given on a five-point scale. Items that ask about negative perceptions and experiences, such as “How much do you fear the future?” are reverse-coded for scoring. Thus, higher scores for all items indicate better quality of life. The average score for each domain ranged from 4 to 20.

Self-perception of aging was evaluated using the Spanish version of the five-item self-stereotype measure as used in previous studies [24]. Example items include “I am as happy now as I was when I was younger” (yes/no response; reverse coded) and “As I get older, things are (response options: better, the same, worse) than/as I thought they would be”. The first four items are dichotomous, whereas the fifth item uses a three-point Likert scale. For the four dichotomous items, ‘yes’ responses were scored as 1 and ‘no’ responses as 0, and scores for the two negatively worded items were reverse-coded. For the three-category item, responses were scored as 0 (‘worse’), 1 (‘the same’), and 2 (‘better’). A mean score was then calculated across the five items, yielding a global index ranging from 0 to 1.6, with higher scores indicating a more positive self-perception of aging. This scale has been previously used in studies conducted in Spain [28,31] and showed acceptable internal consistency in the present study (*α* = 0.72). Given its brevity and the predominance of dichotomous items, the measure was treated as a global index, and the mean score was used as a single observed indicator of self-perception of aging in the PLS-SEM models.

To assess age-related stereotypes, we used two versions of the Stereotype Content Model Scale [22]. One version assessed cultural perception of ageing stereotypes (“To what extent people in Spain believe that Older Adults are…?”), whereas the other assessed self-stereotypes (“To what extent these traits describe you…?”). Items were rated in a Likert-type scale ranging from 1 (not at all) to 7 (very much). Both versions included two dimensions: competence (e.g., self-confident, intelligent) and warmth (e.g., friendly, tolerant), and showed good reliability indices across the four factors. In the structural models, cultural age-related stereotypes of competence and warmth were specified as exogenous latent constructs. In contrast, self-stereotypes of competence and warmth were specified as mediating latent constructs, each measured by its corresponding Stereotype Content Model items. These constructs were modelled as predictors of self-perception of aging and, indirectly, of HRQoL outcomes.

Lastly, the survey included a section to collect self-reported sociodemographic information (e.g., marital status, education level).

#### 2.2.2. Clinical Health Data

Data on NAEs occurring during the previous two years were retrospectively extracted from the CoRIS database. These data were clinician-reported rather than self-reported by participants. NAEs were categorized into eight areas: Metabolic, Neurological, Bone, Neoplastic, Hepatic, Renal, Cardiovascular, and Other. The response was dichotomous (0 = absence; 1 = presence). A composite variable, NAEs, was created by summing these categories (0 = no events; 8 = all events). This composite NAEs index reflects the overall burden of distinct non-AIDS comorbidities rather than a reflective latent construct and was therefore treated as a single observed indicator of comorbidity burden in the PLS-SEM models.

### 2.3. Data Analysis

First, we conducted descriptive analyses, examined bivariate correlations, and comparison scores between men and women. Second, we used Partial Least Squares Structural Equation Modeling (PLS-SEM) as a variance-based technique to explore and predict the relationships among NAEs, cultural age-related stereotypes, self-perception of aging, and HRQoL. In line with recommendations for PLS-SEM, which is particularly suitable for exploratory models, relatively small samples and prediction-oriented objectives [32], our main goal was to explain variance in the endogenous constructs rather than to test a strictly confirmatory covariance structure.

To assess the statistical power of the model, a post hoc power analysis was conducted using G*Power 3.1 [33]. The analysis focused on the main endogenous variable in the model using the Multiple Linear Regression family (*F* tests; linear multiple regression: fixed model, *R*^2^ deviation from zero). For the specific path from self-perception of aging to general health, which exhibited an effect size of *f*^2^ = 0.228, and considering a total sample size of *N* = 285, four predictors, and a significance level of α = 0.05, the analysis yielded a statistical power (1 − β) of 0.99. This indicates very high power to detect the observed effect and a very low probability of Type II error for this association, suggesting that the sample size was adequate for the main paths of interest.

In the structural models, NAEs and cultural age-related stereotypes of competence and warmth were specified as exogenous predictors, self-stereotypes of competence and warmth and self-perception of aging as mediating variables, and HRQoL (general health and the six WHOQOL-HIV-BREF domains) as endogenous outcomes. Cultural age-related stereotypes, self-stereotypes, and HRQoL dimensions were modelled as reflective latent constructs measured by their corresponding items. In contrast, NAEs and self-perception of aging were treated as single-indicator composites reflecting overall comorbidity burden and a brief global index of aging self-perception, respectively.

The analysis followed a two-step procedure. In the first step, we analyzed the relationships between the number of NAEs during the two-year retrospective period and the HRQoL dimensions. Additionally, the relationships between cultural age stereotypes (competence and warmth) and HRQoL dimensions were examined through self-perceived competence and warmth. In the second step, a mediation analysis was conducted including self-perception of aging as a subsequent mediator in the model linking these antecedents and the HRQoL dimensions.

Measurement model evaluation focused on internal consistency reliability (Cronbach’s α and composite reliability) and convergent validity (Average Variance Extracted, AVE). Structural model evaluation focused on standardized path coefficients, their significance (bootstrapping with 5000 resamples), the explained variance (*R*^2^) of endogenous constructs and collinearity diagnostics (inner variance inflation factors, VIFs), with all VIF values < 2. Data were analyzed using SPSS 27 [34] and SmartPLS v3.0 [35].

## 3. Results

### 3.1. Participants’ Characteristics

The final sample included 285 PLHIV aged 50 years or older. The sample was predominantly composed of men and included individuals already engaged in hospital-based HIV care, with a high proportion of participants presenting an undetectable viral load. The mean age was approximately 57. Most participants had completed elementary or high school education, were employed, and reported low-to-medium socioeconomic status. Table 1 summarizes the sociodemographic and clinical characteristics of the sample, including age, sex, socioeconomic status, duration of HIV infection, antiretroviral treatment, and viral suppression.

### 3.2. Descriptive Analyses and Bivariate Associations

Given that each participant assessed both cultural stereotypes and their own self-perceptions in terms of warmth and competence (i.e., a within-subjects design), repeated-measures ANOVAs were conducted. The analyses revealed significant differences between cultural perceptions of competence and warmth in older adults. Specifically, participants attributed higher levels of warmth (*M* = 5.18; *SD* = 1.11) than competence (*M* = 5.07; *SD* = 1.04) to older adults in Spain, *F*(1, 284) = 4.206, *p* = 0.041, η^2^ = 0.015. A second repeated-measures ANOVA revealed a substantially larger difference in participants’ self-descriptions, *F*(1, 284) = 137.212, *p* < 0.001, η^2^ = 0.33, indicating that participants described themselves as significantly higher in warmth (*M* = 6.11; *SD* = 0.79) than in competence (*M* = 5.57; *SD* = 0.92). Taken together, these analyses suggest that the warmth–competence difference is much larger in self-descriptions than in perceptions of older adults, as reflected in η^2^ = 0.33 > η^2^ = 0.015.

Comparisons between male and female participants showed significant differences in the HRQoL level of independence domain and in self-perception of aging. Men scored higher than women in level of independence (male: *M* = 15.75, *SD* = 3.07; female: *M* = 14.61, *SD* = 3.19); *t*(281) = 2.617, *p* = 0.009; Cohen’s *d* = 0.38). Regarding self-perception of aging, a significant difference was also observed, with men reporting more positive self-perceptions of aging than women (male: *M* = 0.69, *SD* = 0.37; female: *M* = 0.58, *SD* = 0.37; *t*(281) = 1.880, *p* = 0.031, *d* = 0.27).

Descriptive statistics and correlations for all study variables are presented in Table 2, which shows the pattern of associations among stereotype measures, self-perception of aging, NAEs, and the HRQoL dimensions. Cultural age-related stereotypes of competence and warmth were positively correlated with self-competence and self-warmth, respectively. Self-perception of aging was positively correlated with all HRQoL dimensions and negatively correlated with the number of NAEs. Age was not significantly correlated with stereotype measures, self-perception of aging, or NAEs.

### 3.3. Model Testing

We followed a two-step analysis in accordance with the guidelines on partial least squares structural modeling (PLS-SEM) provided by Hair et al. [32]. In the first step, we assessed the measurement models (tests of the measures’ validity and reliability). Secondly, we evaluated the structural model.

#### 3.3.1. Measurement Models

The measurement models showed significant relationships between most indicators and their corresponding latent constructs (*p* < 0.01; except for NAEs and self-perception of aging, which were specified as observed composite variables represented by a single indicator: NAEs were modelled as a comorbidity burden index based on the count of the eight NAEs collected in the database, and self-perception of aging as a brief global index derived from a five-item scale with predominantly dichotomous responses). Most outer loadings were above the recommended value of 0.70, although some indicators showed lower loadings. Following the predefined measurement strategy, the physical health domain (one item: degree to which physical problems related to HIV infection are bothersome), the level of independence domain (one item: degree to which medical treatment is needed to function in daily life), and the environmental health domain (two items: satisfaction with access to health services and satisfaction with transport services) were refined by removing indicators with low factor loadings (i.e., below 0.50).

Table 3 reports the outer loadings, composite reliability, Cronbach’s alpha, and AVE values for all constructs. The Composite Reliability (r_c_) values were adequate for all constructs, indicating satisfactory internal consistency [30]. However, Cronbach’s alpha values were slightly below the conventionally recommended thresholds for physical health (α = 0.65) and spirituality, religion and personal beliefs (α = 0.61). AVE values were above the critical threshold of 0.50 except for the environmental health domain, which showed an AVE of 0.46. Although the AVE value for this construct was slightly below the recommended threshold of 0.50, the construct was retained in the model because the remaining reliability indicators demonstrated acceptable values. Specifically, composite reliability and Cronbach’s alpha exceeded the recommended thresholds, indicating adequate internal consistency. Furthermore, this dimension has been included in previous studies using similar conceptualizations; therefore, eliminating the construct would hinder comparability with prior research and reduce continuity in the empirical literature in this field. Removing additional items solely to increase the AVE could also compromise the construct’s theoretical content validity.

#### 3.3.2. Structural Model

**Figure 1:**
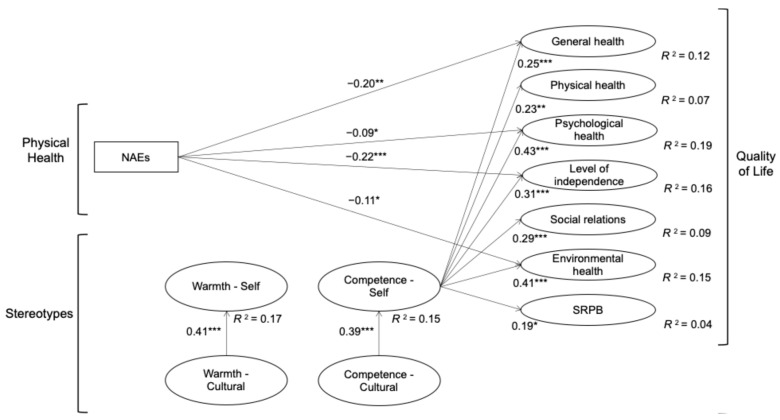
Structural model: Direct relationships. Note: NAEs: Number of non-AIDS health events 2 years before. SRPB: Spirituality, religion and personal beliefs. * *p* < 0.05. ** *p* < 0.01. *** *p* < 0.001.

Mediation analysis was analyzed in two steps. The first step analyzed that general health and the HRQoL dimensions could be predicted by the antecedents. In the direct-effects model, self-perceived competence was significantly associated with all HRQoL dimensions, whereas self-perceived warmth was not significantly associated with HRQoL outcomes. The number of NAEs was negatively associated with general health, psychological health, environmental health, and level of independence. Overall, the explained variance of the HRQoL dimensions in this model was low. Figure 1 displays the direct-effects of the PLS-SEM model, including cultural stereotypes, self-stereotypes, NAEs, and HRQoL, with standardized path coefficients and explained variance (*R*^2^) for each outcome.

**Figure 2:**
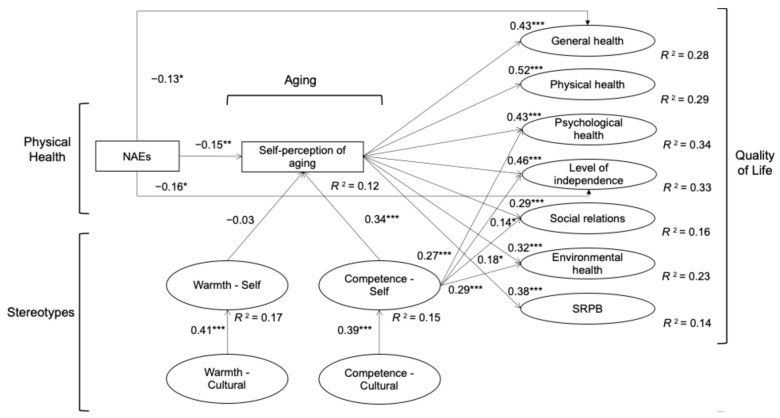
Structural model: General health and WHOQOL dimensions. Note: NAEs: Number of non-AIDS health events 2 years before. SRPB: Spirituality, religion and personal beliefs. * *p* < 0.05. ** *p* < 0.01. *** *p* < 0.001.

The second step involved including self-perception of aging as a mediating variable in the model. In this mediation model, all inner VIF were below 2. The model explained 12.1% of the variance in self-perception of aging and between 14% and 34% of the variance in the different HRQoL dimensions. Most structural path coefficients were statistically significant, except for the path from self-perceived warmth to self-perception of aging and several direct paths included in the initial direct-effects model. Figure 2 shows the final mediation model, in which self-perception of aging mediates the associations between NAEs, self-stereotypes, and the different HRQoL domains, together with the corresponding standardized path coefficients and *R*^2^ values.

The number of NAEs in the two-year retrospective period was negatively associated with self-perception of aging. Cultural age stereotypes of competence and cultural warmth were positively associated with their respective self-stereotype dimensions. Only self-perceived competence was positively associated with self-perception of aging. In turn, self-perception of aging was positively associated with all HRQoL dimensions. Standardized path coefficients were higher for general health, physical health, psychological health, and level of independence than for social relations, environmental health, and spirituality, religion, and personal beliefs.

Additional mediation analysis revealed several significant indirect effects (Table 4). Specifically, the number of NAEs in the two-year retrospective period displayed significant indirect and negative effects on general health and all HRQoL dimensions, mediated by self-perception of aging. Cultural age stereotypes of competence exerted a significant indirect effect on several HRQoL domains (psychological health, level of independence, social relations, and environmental health) through self-perceived competence. Furthermore, sequential multi-step mediation pathways were significant, indicating that cultural age stereotypes of competence indirectly affected general health and all HRQoL dimensions via the sequential mediators of self-perceived competence and self-perception of aging. Similarly, self-perceived competence maintained significant indirect effects on general health and all HRQoL dimensions through the mediation of self-perception of aging.

## 4. Discussion

To our knowledge, this is among the first studies in PLHIV to examine, within a single analytical model, the relationships among comorbidities (operationalized as NAEs), cultural age-related stereotypes, self-perception of aging, and HRQoL. The findings suggest that self-perception of aging occupies a central position in these associations. Specifically, a higher number of NAEs was associated with a less positive self-perception of aging. In contrast, a more positive self-perception of aging was associated with better scores across all HRQoL dimensions. In addition, although both cultural age-related stereotypes dimensions were related to their corresponding self-stereotypes, only self-perceived competence showed a positive association with self-perception of aging.

These findings are consistent with the conceptual framework proposed by Stereotype Embodiment Theory, which posits that culturally age-related stereotypes may become internalized and influence health through self-perceptions of aging [25]. In our study, the competence dimension appeared to be more relevant than warmth in the pathway linking stereotypes with HRQoL, suggesting that not all age-related stereotypes have the same implications for how older adults evaluate their own aging. This pattern is also coherent with previous work showing that competence-related attributions may be especially important in shaping behaviors, expectations, and care processes in older populations [36,37].

The present results also suggest the coexistence of two complementary pathways influencing HRQoL in older PLHIV. On the one hand, there is a clinical pathway in which NAEs were negatively associated with self-perception of aging and several HRQoL dimensions. On the other hand, there is a psychosocial pathway, in which culturally shared age stereotypes, particularly competence-related stereotypes, were linked to self-perception of aging through their internalization into the self-concept. This psychosocial pathway is consistent with prior work showing that older PLHIV often experience multiple forms of stigma and ageism, and that these experiences are associated with poorer mental health and wellbeing [18,19]. In addition, reviews of healthy aging in older women with HIV have highlighted the central role of psychosocial factors, including stigma, social support and psychological resilience, in shaping quality of life and aging outcomes [20]. These findings reinforce the idea that stereotype internalization and self-perception of aging are plausible mechanisms through which social attitudes and psychosocial stressors may influence HRQoL in this population. Together, these findings support the view that aging with HIV should not be understood exclusively in biomedical terms, but also in relation to psychosocial processes that shape how individuals experience aging and evaluate their quality of life.

Self-perception of aging was positively associated with all HRQoL dimensions, with stronger coefficients for general health, physical health, psychological health, and level of independence. This broad pattern is consistent with previous evidence showing that more positive views on aging are associated with better health, functioning, and wellbeing in later life. In the context of HIV, this issue may be particularly relevant because people aging with HIV face the combined influence of comorbidity, long-term treatment exposure, stigma, and social disadvantage, all of which may affect both the experience of aging and HRQoL [4,13,14,15,16,17]. At the same time, previous research has reported more modest or domain-specific effects of psychosocial interventions and aging-related beliefs on psychological outcomes among PLHIV, and in some cases, these effects are attenuated or non-significant, with low-quality and heterogeneous evidence [38]. This heterogeneity suggests that the impact of self-perceptions of aging and stereotypes may vary across contexts, measures and subgroups, and that the present findings should be interpreted as part of a broader and still-evolving evidence base.

An additional finding was that chronological age itself was not significantly associated with stereotype measures, self-perception of aging, or retrospective NAEs in this sample of older PLHIV. It suggests that, once people have reached older age with HIV, subjective and social dimensions of aging—such as self-perceptions of aging and internalized stereotypes—may be more informative than chronological age for understanding differences in HRQoL. We also observed some sex differences in self-perception of aging and level of independence, although these findings should be interpreted cautiously because women were underrepresented in the sample. Previous studies on sex differences in self-perceptions of aging have yielded mixed results. A cross-national study (*N* = 5566 older adults across 20 countries) did not find gender differences [39], whereas other studies have indicated that females score higher in self-perception of aging and positive aging experience, and emotional domains, but not in physical and engagement domains [40]. Further research in more balanced HIV samples is needed.

From a clinical and public health perspective, the findings suggest that improving the wellbeing of older PLHIV may require action at both the clinical and psychosocial levels. First, prevention and management of comorbidities remain essential, given the negative associations observed between NAEs and both self-perception of aging and several HRQoL dimensions. Second, interventions aimed at promoting more positive self-perceptions of aging and challenging age-related beliefs among older adults with HIV (e.g., through psychoeducational or group-based programs) may represent a complementary strategy to improve HRQoL. Such an approach is in line with previous work emphasizing that psychosocial stressors, including HIV-related stigma, ageism, social isolation, and low resilience, are key determinants of mental health and quality of life in older PLHIV [18,19].

It is also important to emphasize the need to move towards a model of care for PLHIV adapted to chronic conditions, in which the patient is at the center, and that incorporates attention to cultural stereotype perception in this process. For example, Fernandez-Ballesteros et al. have pointed out that the effectiveness of person-centered therapy depends on a greater perception of competence towards older adults in care centers [36]. Research has also shown that interventions aimed at changing self-perception of aging can be effective, as they improve health behaviors and consequently functional health [41]. In addition, interventions aimed at training healthcare providers to reduce ageist and HIV-related stereotypes and foster more competence-affirming interactions with older patients may be useful, as suggested by recommendations to increase the cultural competence and sensitivity of professionals caring for older PLHIV [42]. Community-level initiatives aimed at reducing stigma and ageism in the broader social environment may also help to reduce the internalization of negative stereotypes and support better HRQoL in this population, in line with calls to address HIV-related stigma through community education and support programs [43,44]. Addressing all these factors alongside comorbidity management may therefore be necessary to achieve truly person-centered and “successful” aging with HIV. This approach is compatible with current calls to move HIV care beyond viral suppression toward person-centered models focused on long-term wellbeing and quality of life [5,6,8,9,45].

### Limitations

Several limitations should be considered when interpreting these findings. First, participants were recruited by convenience sampling from hospital-based HIV care settings within the CoRIS cohort, which may have resulted in a sample that was more clinically stable and more engaged in care than the broader population of older PLHIV [45,46]. CoRIS does not include people who survived from earlier stages of the epidemic before the cohort was established, which may underrepresent long-term survivors with greater cumulative morbidity burden [46]. Women represented 21.4% of the sample, which is similar to national estimates, indicating that women account for approximately 15–20% of PLHIV in Spain [3]. However, the absolute number of women was limited, reducing statistical power for sex-specific analyses. The sample also shows very high levels of viral suppression, which may underrepresent people who are less regularly linked to care. However, national cascade-of-care data suggest that high rates of treatment uptake and viral suppression are now common in Spain [47].

Second, this article reports a secondary analysis restricted to participants with two years of retrospective clinical data in CoRIS, which reduced the final sample size. A longer retrospective period was considered, but a 3-year window would have led to a further loss of participants because some had entered the cohort more recently, and some centres lacked complete reporting for earlier years. The 2-year retrospective window was therefore selected as a pragmatic and clinically meaningful period to capture recent NAEs while retaining an adequate sample size for the analyses. However, this relatively short horizon may not fully reflect longer-term trajectories of comorbidity and aging-related experiences. In addition, the composite NAEs index did not allow us to examine the differential impact of specific comorbidity types or their severity. It may oversimplify the heterogeneity of clinical profiles among older PLHIV. Both NAEs and self-perception of aging were modelled as observed composite variables represented by single indicators, and the self-perception of aging measure was very brief with a limited response range, which may limit the depth of measurement and the assessment of more nuanced latent dimensions. We also lacked information on several lifestyle and behavioural factors (e.g., smoking, alcohol use, adherence) and psychosocial variables (e.g., stigma, loneliness, resilience, social support) that may influence both health events and HRQoL.

Third, because participants were recruited by their treating clinicians and completed the survey within hospital settings, some degree of selection and social desirability bias cannot be ruled out. However, questionnaires were self-administered in private spaces and did not collect identifying information. Finally, the study used PLS-SEM in an exploratory, prediction-oriented framework appropriate for relatively complex models and moderate sample sizes [32]. The explained variance for some HRQoL dimensions should be interpreted as small-to-moderate, indicating that additional clinical and psychosocial factors not included in the model are also important. The removal of several items from some HRQoL domains due to low factor loadings, along with the environmental health dimension not meeting the minimum recommended AVE threshold, suggests that results for these variables should be interpreted with caution. Overall, the findings should be viewed as evidence of relevant associations rather than as a fully explanatory model of HRQoL in older PLHIV, and future studies in larger and more diverse samples with longer follow-up are needed to replicate and extend these results.

## 5. Conclusions

In conclusion, this study shows that, among older PLHIV, self-perception of aging is closely associated with HRQoL and may partially mediate the negative association between non-AIDS comorbidities and some HRQoL dimensions. The findings also suggest that cultural age-related stereotypes, particularly the competence dimension, influences quality of life indirectly through its internalization and its impact on self-perception of aging. The perception of self-competence is associated with a more positive self-perception of aging, which in turn relates to better quality of life.

These results support a broader understanding of aging with HIV that includes both biomedical and psychosocial processes. Alongside the prevention and management of comorbidities, interventions aimed at promoting more positive self-perceptions of aging and addressing negative competence-related stereotypes may help improve long-term well-being and person-centered HIV care. Future longitudinal studies with larger and more diverse samples should further examine these pathways and evaluate their implications for clinical practice and healthy aging in PLHIV.

## Figures and Tables

**Table 1:** Sociodemographic and clinical characteristics (*N* = 285).

Sociodemographic and Clinical Variables		% (*n*)
Gender	Male	77.9 (222)
	Female	21.4 (61)
	Transgender	0.7 (2)
Education level	No studies	7.0 (20)
	Elementary School	39.3 (112)
	High School	28.8 (82)
	University degree	24.6 (70)
	Other	0.3 (1)
Work situation	Employee	51.2 (146)
	Unemployed	25.6 (73)
	Retired	9.1 (26)
	Impaired	13.0 (37)
	Not answer	1.1 (3)
Socioeconomic status	Low	17.2 (49)
	Medium-Low	46.6 (133)
	Medium-High	34.0 (97)
	High	2.1 (6)
Marital status	Married or living together	41.1 (117)
	Divorced or separated	17.5 (50)
	Single	35.4 (101)
	Widowed	5.6 (16)
	Not answer	0.4 (1)
Ethnic group	Caucasian/European	81.7 (233)
	Hispanic/Latin American	16.5 (47)
	Asian	0.4 (1)
	African	0.7 (2)
	Other/Not answer	0.7 (2)
Sexual behavior	Heterosexual	49.5 (141)
	Homosexual	35.4 (101)
	Bisexual	10.5 (30)
	No answer	4.6 (13)
Health status	Very bad	0.7 (2)
	Bad	3.5 (10)
	Neither bad nor good	21.1 (60)
	Good	58.6 (167)
	Very good	16.1 (46)
Currently sick	Yes	17.9 (51)
	No	75.8 (216)
	No answer	6.3 (18)
Age, years, mean (*M* ± *SD*)		57.4 ± 7.1 [49–83 years old]
Duration of infection, years, mean (*M* ± *SD*)		8.86 ± 5.7 [Min = 1; Max = 35]
Years taking antiretroviral therapy (*M* ± *SD*)		7.2 ± 3.8 [Min = 1; Max = 26]
Undetectable plasma viral load		95.8 (273)

Note: Data in percentages (number of participants between brackets) unless otherwise stated.

**Table 2:** Descriptive statistics and correlations between study variables.

	*M*	*SD*	1	2	3	4	5	6	7	8	9	10	11	12	13
1. Competence—Cultural stereotype	5.07	1.04	1												
2. Warmth—Cultural stereotype	5.18	1.11	0.59 ***	1											
3. Competence—Self-stereotype	5.57	0.92	0.38 ***	0.37 ***	1										
4. Warmth—Self-stereotype	6.11	0.79	0.37 ***	0.40 ***	0.60 ***	1									
5. Self-perception of aging	0.67	0.37	0.20 ***	0.10	0.33 ***	0.18 **	1								
6. WHOQOL—General health	14.82	3.29	0.10	0.13 *	0.28 ***	0.20 ***	0.50 ***	1							
7. WHOQOL—Physical health	15.84	3.13	0.15 *	0.14 *	0.22 ***	0.15 **	0.49 ***	0.52 ***	1						
8. WHOQOL—Psychological health	15.13	2.82	0.12 *	0.11	0.41 ***	0.24 ***	0.52 ***	0.65 ***	0.53 ***	1					
9. WHOQOL—Level of independence	15.51	3.12	0.13 *	0.17 **	0.36 ***	0.26 ***	0.50 ***	0.55 ***	0.65 ***	0.57 ***	1				
10. WHOQOL—Social relations	14.28	3.46	0.08	0.07	0.25 ***	0.15 **	0.36 ***	0.52 ***	0.39 ***	0.67 ***	0.38 ***	1			
11. WHOQOL—Environmental health	15.84	2.24	0.10	0.16 **	0.34 ***	0.21 ***	0.38 ***	0.59 ***	0.47 ***	0.60 ***	0.49 ***	0.65 ***	1		
12. WHOQOL—SRPB	14.40	3.92	0 0.15 *	−0.00	0.18 **	0.06	0.43 ***	0.33 ***	0.34 ***	0.52 ***	0.22 ***	0.50 ***	0.33 ***	1	
13. Non-AIDS clinical events (NAEs)	0.29	0.60	−0.01	−0.12 *	−0.06	−0.06	−0.17 **	−0.21 ***	−0.13 *	−0.12 *	−0.18 **	−0.12	−0.16 **	−0.02	1
14. Age	57.38	7.15	0.04	0.08	0.03	−0.01	−0.06	−0.01	0.02	−0.01	−0.15 *	−0.06	0.05	0.15 *	0.10

Note. SRPB: Spirituality, religion and personal beliefs. * *p* < 0.05. ** *p* < 0.01. *** *p* < 0.001. The items in scales 1–5 have a range of 7 points and 6–12 of 5 points.

**Table 3:** Measurement model assessment: Outer loadings, reliability, and average variance extracted.

Latent Variable	Item	λ	r_c_	a	AVE
NAEs	Number of non-AIDS clinical events 2 years before	1.00	1.00	1.00	1.00
Self-perception of aging		1.00	1.00	1.00	1.00
Competence—self			0.86	0.79	0.55
	Competence	0.75			
	Self-confidence	0.84			
	Independence	0.66			
	Competitive	0.72			
	Intelligent	0.72			
Competence—cultural			0.87	0.82	0.58
	Competence	0.77			
	Self-confidence	0.81			
	Independence	0.67			
	Competitive	0.77			
	Intelligent	0.78			
Warmth—self			0.85	0.76	0.58
	Tolerant	0.71			
	Warmth	0.86			
	With good intentions	0.81			
	Sincere	0.66			
Warmth—cultural			0.90	0.84	0.68
	Tolerant	0.81			
	Warmth	0.85			
	With good intentions	0.85			
	Sincere	0.80			
WHOQOL—General health			0.90	0.77	0.81
	QOL	0.88			
	Satisfaction	0.92			
WHOQOL—Physical health			0.81	0.65	0.59
	Pain and discomfort	0.68			
	Energy and fatigue	0.85			
	Sleep and rest	0.76			
WHOQOL—Psychological health			0.87	0.82	0.54
	Positive feelings	0.80			
	Concentration ability	0.60			
	Bodily image self-acceptance	0.64			
	Self-satisfaction	0.83			
	Negative feelings	0.68			
	Religion, spirituality and personal beliefs (personal life meaning)	0.82			
WHOQOL—Level of independence			0.86	0.76	0.68
	Mobility	0.60			
	Activities of daily living	0.91			
	Work capacity	0.93			
WHOQOL—Social relations			0.88	0.82	0.65
	Social inclusion	0.77			
	Personal relationships	0.87			
	Sexual satisfaction	0.76			
	Social support	0.81			
WHOQOL—Environmental health			0.83	0.76	0.46
	Physical safety and security	0.76			
	Physical environment	0.63			
	Financial resources	0.65			
	Information for daily living	0.62			
	Participation in leisure activities	0.74			
	Home environment	0.63			
WHOQOL—Spirituality, religion and personal beliefs			0.79	0.61	0.56
	Forgiveness and blame	0.62			
	Concerns about the future	0.90			
	Death and dying	0.71			

Note. λ = outer loading. r_c_ = composite reliability. a = Cronbach’s alpha. AVE = Average Variance Extracted. NAEs and self-perception of aging were specified as single-indicator composite variables; for these constructs, reliability and AVE are fixed at 1.00 by definition in the PLS-SEM specification.

**Table 4:** Specific indirect effects.

			Confidence Interval	
Mediation Path	Original Sample (b)	*p*-Value	LLCI (Lower Limit)	ULCI (Upper Limit)
NAEs → Self-perception of aging → General health	−0.065	0.008	−0.113	−0.023
NAEs → Self-perception of aging → Physical health	−0.078	0.008	−0.131	−0.026
NAEs → Self-perception of aging → Psychological health	−0.065	0.009	−0.114	−0.022
NAEs → Self-perception of aging → Level of independence	−0.068	0.007	−0.117	−0.024
NAEs → Self-perception of aging → Social relations	−0.044	0.014	−0.082	−0.016
NAEs → Self-perception of aging → Environmental health	−0.049	0.012	−0.087	−0.017
NAEs → Self-perception of aging → SRPB	−0.058	0.016	−0.106	−0.018
Competence—Cultural → Competence—Self → Psychological health	0.106	0.002	0.053	0.171
Competence—Cultural → Competence—Self → Level of independence	0.055	0.042	0.009	0.114
Competence—Cultural → Competence—Self → Social relations	0.071	0.019	0.017	0.128
Competence—Cultural → Competence—Self → Environmental health	0.113	0.001	0.061	0.175
Competence—Cultural → Competence—Self → Self-perception of aging	0.133	0.000	0.076	0.203
Competence—Cultural → Competence—Self → Self-perception of aging → General health	0.058	0.001	0.032	0.093
Competence—Cultural → Competence—Self → Self-perception of aging → Physical health	0.069	0.001	0.039	0.109
Competence—Cultural → Competence—Self → Self-perception of aging → Psychological health	0.058	0.001	0.032	0.092
Competence—Cultural → Competence—Self → Self-perception of aging → Level of independence	0.061	0.001	0.035	0.095
Competence—Cultural → Competence—Self → Self-perception of aging → Social relations	0.039	0.004	0.020	0.069
Competence—Cultural → Competence—Self → Self-perception of aging → Environmental health	0.043	0.002	0.023	0.073
Competence—Cultural → Competence—Self → Self-perception of aging → SRPB	0.051	0.002	0.027	0.084
Competence—Self → Self-perception of aging → General health	0.146	0.000	0.094	0.210
Competence—Self → Self-perception of aging → Physical health	0.175	0.000	0.113	0.243
Competence—Self → Self-perception of aging → Psychological health	0.146	0.000	0.093	0.207
Competence—Self → Self-perception of aging → Level of independence	0.154	0.000	0.101	0.217
Competence—Self → Self-perception of aging → Social relations	0.099	0.000	0.058	0.155
Competence—Self → Self-perception of aging → Environmental health	0.109	0.000	0.066	0.164
Competence—Self → Self-perception of aging → SRPB	0.130	0.000	0.077	0.192

Note: NAEs: Number of non-AIDS clinical events 2 years before. SRPB: Spirituality, religion and personal beliefs.

## Data Availability

The data supporting the findings of this study may be requested from the corresponding author. Public access is not possible due to privacy restrictions.

## Abbreviations

The following abbreviations are used in this manuscript:

AIDS	Acquired Immune Deficiency Syndrome
ART	Antiretroviral Therapy
AVE	Average Variance Extracted
HIV	Human Immunodeficiency Virus
HRQoL	Health-Related Quality of Life
NAEs	Number of Non-AIDS Health Events 2 Years Before
PLHIV	People Living with HIV
SRPB	Spirituality, Religion, and Personal Beliefs
VIF	Variance Inflation Factors
